# The supplementary therapeutic DMARD role of low-dose glucocorticoids in rheumatoid arthritis

**DOI:** 10.1186/ar4685

**Published:** 2014-11-13

**Authors:** Maurizio Cutolo, Cornelia M Spies, Frank Buttgereit, Sabrina Paolino, Carmen Pizzorni

**Affiliations:** 1 Research Laboratory and Academic Division of Clinical Rheumatology, Department of Internal Medicine, University of Genova, Viale Benedetto XV 6, 16132 Genova, Italy; 2 Department of Rheumatology and Clinical Immunology, Charité - Universitätsmedizin Berlin, Charitéplatz 1, 10117 Berlin, Germany

## Abstract

The management of rheumatoid arthritis (RA) is primarily based on the use of disease-modifying antirheumatic drugs (DMARDs), mainly comprising synthetic chemical compounds (that is, methotrexate or leflunomide) and biological agents (tumor necrosis factor inhibitors or abatacept). On the other hand, glucocorticoids (GCs), used for decades in the treatment of RA, are effective in relieving signs and symptoms of the disease, but also interfere with radiographic progression, either as monotherapy or in combination with conventional synthetic DMARDs. GCs exert most of their biological effects through a genomic action, using the cytosolic GC receptor and then interacting with the target genes within target cells that can result in increased expression of regulatory - including anti-inflammatory - proteins (transactivation) or decreased production of proinflammatory proteins (transrepression). An inadequate secretion of GCs from the adrenal gland, in relation to stress and inflammation, seems to play an important role in the pathogenesis and disease progression of RA. At present there is clear evidence that GC therapy, especially long-term low-dose treatment, slows radiographic progression by at least 50% when given to patients with early RA, hence satisfying the conventional definition of a DMARD. In addition, long-term follow-up studies suggest that RA treatment strategies which include GC therapy may favorably alter the disease course even after their discontinuation. Finally, a low-dose, modified night-release formulation of prednisone, although administered in the evening (replacement therapy), has been developed to counteract the circadian (night) rise in proinflammatory cytokine levels that contributes to disease activity, and might represent the way to further optimize the DMARD activity exerted by GCs in RA.

## Introduction

Rheumatoid arthritis (RA) is a multifactorial, chronic inflammatory and immune-mediated syndrome that causes joint damage, but can in selected patients present with different tissue and organ involvement [1]. Following the 2010 American College of Rheumatology/European League Against Rheumatism RA classification criteria, an overall score ≥6/10 is needed for classification of a patient as having RA [2]. However, these criteria should only be used if a clinical case of RA is likely; namely the patient should have at least one joint with a definite clinical synovitis, not explained by another disease.

The sensitivity of these criteria was recently measured to be higher than its precursor of 1987 while having a lower specificity [3]. Notably, in RA chronic synovial tissue inflammation and hyperplasia drive articular destruction and bone erosion, leading to functional decline and disability [4].

Biological disease-modifying antirheumatic drugs (DMARDs) target particular soluble extracellular mediators (that is, cytokines) or cell surface molecules (that is, CD20 or CD86) with high specificity [5]. Conversely, conventional synthetic DMARDs usually act within cells, but nonetheless may also have specific targets such as that designed to target Janus kinases and constituting the first targeted synthetic DMARD, named tsDMARD, following a proposed new nomenclature [6].

On the other hand, glucocorticoids (GCs), used for decades in the treatment of RA, are effective in relieving signs and symptoms of the disease and also interfere with radiographic progression, either as monotherapy or in combination with synthetic DMARDs [7]. An inadequate secretion of GCs from the adrenal gland, in relation to stress and inflammation, seems to play an important role in the pathogenesis and disease progression of RA [7]. As a matter of fact, in the most recent European League Against Rheumatism (EULAR) recommendations for the management of RA, low-dose GCs have been confirmed as at least part of the initial treatment strategy (in combination with one or more conventional synthetic DMARDs) for at least 6 months [8].

## Understanding the anti-inflammatory actions of glucocorticoids

Despite being among the most effective anti-inflammatory treatments for chronic inflammatory diseases, the mechanisms by which GCs effect repression of inflammatory gene expression remain only incompletely understood. Direct interaction of the GC receptor (nuclear receptor subfamily 3, group C, member 1 (NR3C1)) with inflammatory transcription factors to repress transcriptional activity - that is, transrepression - represents one mechanism of action. However, transcriptional activation - or transactivation - by the GC receptor (NR3C1) also represents an important mechanism of GC action. In addition, GCs rapidly and profoundly increase expression of multiple genes, many with properties consistent with the repression of inflammatory gene expression [9]. On the other hand, RNA-binding proteins and microRNA play an important role in the pathophysiology of chronic inflammation, and seem to have promising value as mechanisms conveying the anti-inflammatory effect of exogenous GCs [10].

**Figure 1:**
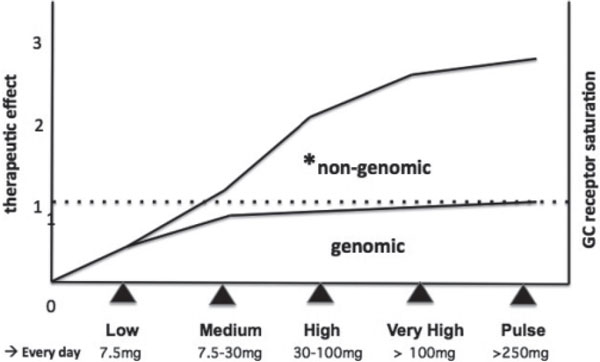
**Relationship between different glucocorticoid doses (prednisone equivalent milligrams), intensity of the therapeutic effect (arbitrary units), and genomic/nongenomic mechanism of action**. Low-dose therapy, <7.5 mg/day; medium-dose therapy, 7.5 up to 30 mg/day; high-dose therapy, 30 up to 100 mg/day; very high-dose therapy, >100 mg/day; pulse therapy, >250 mg/day for a few days [12]. *When administering 100 to 200 mg prednisone per day, all cytosolic glucocorticoid (GC) receptors are occupied (nongenomic action) [23].

In general, GCs provide inhibition of any inflammatory process that seems to be dose dependent, and both a long-term genomic and a short-term nongenomic effect are recognized [11]. Of course, the known side effects of GCs are strongly dose dependent: the longer the therapy or the higher the dose, the more relevant the GC side effects appear [12]. The nomenclature for different GC dosages is reported in Figure 1.

## Genomic action of glucorticoids

As mentioned, and regarding the genomic action, GCs provide most of their effects using the cytosolic glucocorticoid receptor (cGR) - being part of a multiprotein receptor complex also consisting of heat shock proteins and several kinases [13,14].

Since GCs are lipophilic, they are able to pass through the plasma membrane and interact with their distinct receptor within the cells. Of note, the 17-hydroxy, 21-carbon steroid configuration of GCs is the reason for their lipophilicity and the successive receptor binding, and is applicable to both prednisone and prednisolone and other GCs [15].

Following the GC binding to the cGR, the receptor-associated proteins dissociate and the complex of GC/cGR translocates into the nucleus, binding as a homo-dimer to specific DNA binding sites, so-called GC response elements [13]. This genomic action, termed transactivation, leads to the synthesis of anti-inflammatory proteins (such as, for example, annexin (lipocortin) 1, IκB, interleukin (IL)-10) as well as regulatory proteins, involved also in metabolism and various GC side effects. On the other hand, GC/cGR monomers are able to negatively interfere with transcription factors (genomic action termed transrepression) such as nuclear factor-κB, activator protein-1 and nuclear factor for activated T cells, as a consequence reducing the expression of proinflammatory proteins such as IL-1, IL-6 or tumor necrosis factor (TNF) alpha [16,17]. Interestingly, by decreasing TNFα synthesis, GCs probably lead to reduction of inflammatory osteoporosis and joint erosions since TNF physiologically induces the production of receptor activator of nuclear factor-κB ligand that appears to be involved in generation of joint erosions by activating osteoclasts [18].

In conclusion, transrepression and transactivation seem to provide the genomic anti-inflammatory actions exerted especially by low-dose prednisone [19]. A low-dose, modified-release formulation of prednisone, administered in the evening, has been developed to counter the circadian rise in proinflammatory cytokine levels that contributes to disease activity and seems to not interfere with the hypothalamic-pituitary-adrenal (HPA) function [20,21]. Modified-release prednisone might therefore represent the way to further optimize the DMARD activity exerted by low-dose GCs in RA [22].

## Nongenomic action of glucocorticoids

The administration of prednisone, especially at high dosages, induces a rapid clinical improvement that is sometimes too fast to be explained by genomically mediated action, and the presence of nongenomic action is emphasized by the fact that the cGRs are completely occupied when administering 100 to 200 mg/day prednisone [23,24] (Figure 1).

**Figure 2:**
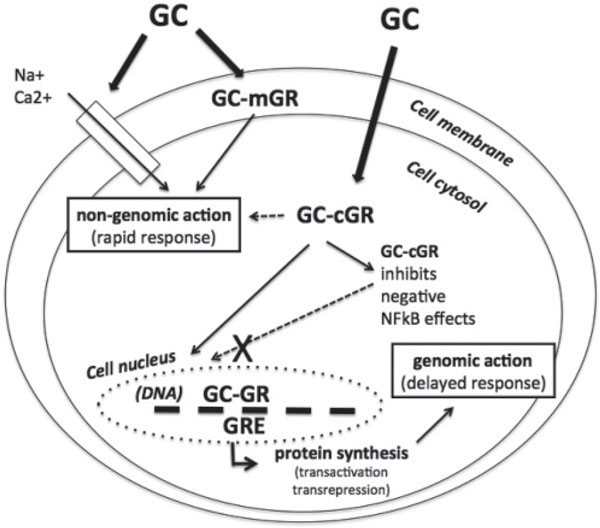
**Representation of major physicochemical glucocorticoid interactions with components of the cellular membrane**. Membrane glucocorticoid receptor (mGR)-mediated nongenomic effects together with cytosolic glucocorticoid receptor (cGR)-mediated genomic effects on nuclear glucocorticoid response elements (GRE) (including inhibition of the nuclear factor-κB complex (NF-κB)) and resultant protein synthesis that can cause increased expression of regulatory - including anti-inflammatory - proteins (transactivation) or decreased production of proinflammatory proteins (transrepression) [16]. GC, glucocorticoid; GR, glucocorticoid receptor.

The proposed nongenomic action for mechanisms that are not cGR-mediated includes their specific interaction with the cGR - resulting, for example, in nongenomic inhibition of arachidonic acid release - and physio-chemical interactions with cellular membranes, resulting in nongenomic compromising of ion cycling across the membranes and cell energy supply, and consequently immune/inflammatory cell function [16,25]. Generally, the rapid nongenomic GC effects that appear quickly, from several seconds to minutes, seem to be obtained by three different mechanisms: physicochemical interactions with components of the cellular membranes (nonspecific nongenomic effects); membrane-bound GC receptor-mediated nongenomic effects; and cGR-mediated nongenomic effects [11,26]. The extraordinary wide range of GC actions can be explained by GC receptor presence in three cell compartments: nucleus, cytoplasm, and plasma membrane (Figure 2).

Whereas the optimization of the genomic action (by low-dose administration) is achieved via GC nighttime availability (modified-release prednisone release around 3:00 a.m.), by respecting the circadian rhythm of both endogenous GC synthesis and night activation of the immune/inflammatory reaction, on the other hand the nongenomic action (high doses) should be obtainable at almost any time of the day when fast effects are required [27-30].

## From the early steps to the more recent evidence for the use of low-dose glucocorticoids in RA

The introduction of GCs for treatment of RA in 1948 and the dramatic clinical responses in a report published in 1949 originated the attribution of the Nobel Prize in Physiology and Medicine to Hench, Kendall and Reichstein. Interestingly, the Mayo Clinic group that discovered GCs recommended as early as 1955 that doses equivalent to 5 to 10 mg/day prednisone should be used in treatment of RA [31]. However, a dose of 20 mg/day prednisolone was used in clinical trials in 1960 (after GCs were available for treatment) to compare with aspirin, and while DMARD properties were well documented in RA patients, the conventional wisdom at that time was that low doses were not disease modifying (that is, did not decrease joint damage as assessed radiographically) [32,33].

Therefore, by the late 1950s, oral GCs were considered appropriate in RA only for severe, potentially life-threatening situations or as short-term bridging therapy while awaiting results of what was regarded as remission-inducing DMARD therapy [34]. However, the first clinical trial involving low-dose GCs was reported in the early 1960s, and compared the evening dose versus the morning dose [34]. The authors stated that their clinical practice of treating all patients with 7.5 mg/day or less was based on 'observations made in the course of studying the phenomenon of morning stiffness' [35,36].

In addition to the clinical efficacy of the low dose, the authors observed that overall 50% of RA patients preferred the evening dose and concluded that low-dose GC therapy (5 mg prednisolone) at night was more efficacious than the morning dose for management of most RA patients.

No clinical trials involving low-dose prednisone were performed until 19 years later when a well-performed, double-blind study was published in the early 1980s [37]. This study was very important since prednisone (5 mg/ day) was taken every morning and added to other drugs in 18 RA patients. Sixteen RA patients were given a placebo in this double-blind study. After 24 weeks, all patients were treated with placebo. Slight functional improvement was noted in the prednisone group during the 24-week period, but deterioration after switching to placebo was sustained for at least 8 weeks. Interestingly, progression of hand erosions occurred in one prednisone-treated patient and in four controls. The authors concluded that low doses of prednisone may be useful as bridge therapy between nonsteroidal anti-inflammatory therapy and use of DMARDs.

Today there is clear evidence that GC therapy, especially long-term, low-dose treatment, slows down radiographic progression by at least 50% when given to patients with early RA, thus satisfying the conventional definition of a DMARD [38]. In addition, long-term follow-up data suggest that treatment strategies which include GC therapy may favorably alter the disease course even after discontinuation of the GCs. Some recent studies such as the Better Anti-rheumatic Farmacotherapy (BARFOT) study, the Utrecht study, the Behandel Strategieen (BeSt) study and the oldest Combination Therapy with RA (COBRA) study have now reported follow-up for more than 2 years [39-48]. More recently, inclusion of low-dose prednisone in a methotrexate-based treatment strategy for tight control in early RA improved patient outcomes [49].

In conclusion, is now possible to state there is clear evidence that low-dose, long-term GC therapy slows radiographic progression by at least 50% in treated early RA patients and therefore GCs exert disease-modifying effects [50-52].

## The supplementary therapeutic DMARD role of low-dose glucocorticoids

Stressful/inflammatory conditions activate the immune system and subsequently the HPA axis through the central and peripheral production of cytokines such as IL-6 and TNFα [53]. A relative adrenal hypofunction, as evidenced by inappropriately normal/low serum cortisol levels and reduced sulfated dehydroepiandrosterone sulphate serum levels, seems to play an important role in the pathogenesis of autoimmune/inflammatory diseases such as RA as well as polymyalgia rheumatica [54,55]. On the other hand, chronic inflammation represents a stimulus for the activation of the stress response system (in particular, adrenal gland) and, over the long term, a subclinical hypofunction of the adrenal glands.

The regular observation of reduced cortisol and adrenal androgen secretion during testing in RA patients not treated with GCs should therefore clearly be regarded as relative adrenal insufficiency in the setting of a sustained inflammatory process, as shown by high IL-6 levels [56]. The circadian changes in the metabolism or nocturnal secretion of endogenous GCs (reduction) observed in RA patients are responsible, in part, for the time-dependent changes that are observed in the inflammatory response and related early morning clinical symptoms of the disease [57].

Melatonin, another circadian nocturnal hormone that is the secretory product of the pineal gland, has been implicated in the time-dependent RA inflammatory reaction with effects that are opposite to those of GC. As a consequence, altered functioning of the HPA axis (early morning reduced cortisol production) and of the pineal gland (night increased melatonin production) found in RA patients seems to be important in the appearance and perpetuation of the clinical circadian symptoms of the disease [57].

Consistently, human proinflammatory cytokine production (for example, TNFα, IL-6, IL-1) exhibits a diurnal rhythmicity with peak levels during the night and early morning, at a time when plasma cortisol is lowest and melatonin levels are highest [58]. In addition, the HPA axis with the major hormone cortisol and the sympathetic nervous system with epinephrine/norepinephrine induce a shift from energy storage to energy utilization by inducing gluconeogenesis, glycogenolysis, and lipolysis [59]. Since both systems are activated during an acute inflammatory process, they serve the body by provision of energy-rich substrates [60].

The clinical and biochemical improvement observed after GC therapy in patients with RA and polymyalgia rheumatica might thus be attributed to a direct dampening of proinflammatory factors as well as to the restoration of the steroid milieu, a sort of GC replacement therapy [61].

## Conclusions

Although the Mayo Clinic group that discovered GCs already recommended in 1955 that doses equivalent to 5 to 10 mg/day prednisone should be used in treatment of RA, much higher dosages have been employed for decades, until a recent reevaluation of low doses. GCs (low dose) during long-term treatment of RA provide most of their effects through a genomic action using the cGR and then interacting with the target genes that can result in increased expression of regulatory - including anti-inflammatory - proteins (transactivation) or decreased production of proinflammatory proteins (transrepression). An inadequate secretion of GCs from the adrenal gland, in relation to chronic inflammation, seems to play an important role in the pathogenesis and disease progression of RA. Long-term follow-up data now suggest that, especially in early RA patients, treatment strategies that include low-dose GC therapy (replacement therapy) - better if respecting the circadian endogenous production rhythm - may favorably alter the disease course, contributing to the DMARD action, and this effect persists even after their discontinuation [62,63].

## Key messages

• The management of RA is primarily based on the use of DMARDs, mainly consisting of synthetic chemical compounds (synthetic DMARDs) and biological agents (biological DMARDs).

• An inadequate secretion of GCs from the adrenal gland, in relation to chronic inflammation, seems to play an important role in the pathogenesis and disease progression of RA [7].

• GC therapy, especially long-term, low-dose treatment (replacement therapy), slows down radiographic progression by at least 50% when given to patients with early RA, hence satisfying the conventional definition of a DMARD.

• GCs low-dose and long-term provide most of their effects through a genomic action using the cGR and then interacting with the target genes that can result in increased expression of regulatory - including anti-inflammatory - proteins (transactivation) or decreased production of proinflammatory proteins (transrepression).

• Several long-term follow-up studies suggest that RA treatment strategies which include GC therapy may favorably alter the disease course even after their discontinuation.

• A low-dose, modified-release formulation of prednisone, administered in the evening, has been developed to counteract the circadian rise in proinflammatory cytokine levels that contributes to disease activity and might represent the way to further optimize the DMARD activity exerted by GCs in RA.

## Abbreviations

cGR: cytosolic glucocorticoid receptor; DMARD: disease-modifying antirheumatic drug; GC: glucocorticoid; HPA: hypothalamic-pituitary-adrenal; IL: interleukin; RA: rheumatoid arthritis; TNF: tumour necrosis factor;

## Competing interests

MC received funds for clinical research from Horizon and Mundipharma. The other authors declare that they have no competing interests.

## Declaration

This article has been published as part of *Arthritis Research & Therapy* Volume 16 Suppl 2, 2014: At the interface between immunology and endocrinology in rheumatic diseases. The full contents of the supplement are available at http://arthritis-research.com/supplements/16/S2.

This supplement was proposed, developed and commissioned by *Arthritis Research & Therapy *and was funded by an educational grant from Horizon Pharma Inc. All published articles were independently prepared by the authors and have undergone peer review in accordance with the journal's standard policies and processes. Horizon Pharma Inc had no input into the topics covered or the articles themselves. The Supplement Editor was appointed by the journal and declares that they have no competing interests.
